# Association of Plasma Heat Shock Protein 70 with Disease Severity, Smoking and Lung Function of Patients with Chronic Obstructive Pulmonary Disease

**DOI:** 10.3390/jcm9103097

**Published:** 2020-09-25

**Authors:** Iva Hlapčić, Andrea Hulina-Tomašković, Marija Grdić Rajković, Sanja Popović-Grle, Andrea Vukić Dugac, Lada Rumora

**Affiliations:** 1 Department of Medical Biochemistry and Haematology, Faculty of Pharmacy and Biochemistry, University of Zagreb, 10000 Zagreb, Croatia; ihlapcic@pharma.hr (I.H.); ahulina@pharma.hr (A.H.-T.); mgrdic@pharma.hr (M.G.R.); 2 Clinical Department for Lung Diseases Jordanovac, University Hospital Centre Zagreb, 10000 Zagreb, Croatia; spopovi1@kbc-zagreb.hr (S.P.-G.); adugac71@gmail.com (A.V.D.); 3 School of Medicine, University of Zagreb, 10000 Zagreb, Croatia

**Keywords:** chronic obstructive pulmonary disease, extracellular heat shock protein 70, smoking, lung function, EDTA plasma

## Abstract

Extracellular heat shock protein 70 (eHsp70) might modulate immune responses in chronic obstructive pulmonary disease (COPD). The aim of the study was to explore eHsp70 concentration in stable COPD, its association with disease severity and smoking status as well as its diagnostic performance in COPD assessment. Plasma samples were collected from 137 COPD patients and 95 healthy individuals, and concentration of eHsp70 was assessed by commercially available enzyme-linked immunosorbent assay (ELISA) kit (Enzo Life Science, Farmingdale, NY, USA). COPD patients were subdivided regarding airflow obstruction severity and symptoms severity according to the Global Initiative for COPD (GOLD) guidelines. eHsp70 concentration increased in COPD patients when compared to controls and increased with the severity of airflow limitation as well as symptoms burden and exacerbation history. eHsp70 concentration did not differ among COPD patients based on smoking status, yet it increased in healthy smokers compared to healthy nonsmokers. In addition, eHsp70 negatively correlated with lung function parameters forced expiratory volume in one second (FEV_1_) and FEV_1_/ forced vital capacity (FVC), and positively with COPD multicomponent indices BODCAT (BMI, airflow obstruction, dyspnea, CAT score), BODEx (BMI, airflow obstruction, dyspnea, previous exacerbations), CODEx (Charlson’s comorbidity index, airflow obstruction, dyspnea, previous exacerbations) and DOSE (dyspnea, airflow obstruction, smoking status, previous exacerbations) With great predictive value (OR = 7.63) obtained from univariate logistic regression, eHsp70 correctly classified 76% of cases. eHsp70 is associated with COPD prediction and disease severity and might have the potential for becoming an additional biomarker in COPD assessment.

## 1. Introduction

Heat shock proteins (Hsps) are highly conserved and ubiquitously expressed proteins that normally act as molecular chaperones which help in the maintenance of protein homeostasis by assisting their folding processes [1,2]. Their involvement in proper protein folding, prevention of protein aggregation and apoptosis is of great importance for cellular function, especially when cells are exposed to stressful conditions [3,4,5]. The 72-kDa Hsp (Hsp70 in the following refers to this protein) is located in the cytosol and nucleus, and its expression is induced as a part of the response to different stressors like heat, bacterial or viral infections [6,7]. However, apart from being an intracellular protein, Hsp70 can be released from cells passively following cellular lysis i.e., necrotic death, and/or actively through nonclassical exocytotic pathways [8,9,10]. When found in the extracellular milieu, Hsp70 becomes a damage-associated molecular pattern (DAMP) and represents a danger signal to the immune system [11].

eHsp70 acts mainly as proinflammatory and activates immune responses by engaging appropriate receptors (toll-like receptors (TLRs) 2 and 4, cluster of differentiation (CD) 14, CD40, CD91, lectin-like oxidized low-density lipoprotein-1 (LOX-1), receptor for advanced glycation end-products (RAGE)) [9,12,13]. On the other hand, eHsp70 might also modulate an adaptive immune response through binding to antigenic peptides and presenting them to antigen-presenting cells [14]. The sources of Hsp70 in peripheral circulation have not been fully elucidated. Still, various viable cells of both hematopoietic (e.g., peripheral blood mononuclear cells) and nonhematopoietic origin (e.g., epithelial cells) are being considered as potential candidates [8,15].

COPD is an inflammatory syndrome characterized by permanent airflow limitation. It is a multicomponent condition with both pulmonary and extrapulmonary effects [16]. Chronic respiratory inflammation involves activation and infiltration of macrophages and neutrophils and leads to abnormal immune responses, mucus hypersecretion, oxidant-antioxidant imbalance and apoptosis [17]. The role of Hsp70 in COPD pathogenesis is still unclear, despite the efforts of some researchers. It was found that Hsp70 is increased in sputum of COPD patients compared to both healthy smokers and nonsmokers [18]. In addition, increased expression of Hsp70 at both the mRNA and protein level was detected in lung tissue of COPD patients [17]. To the contrary, lower numbers of Hsp70 immunoreactive cells in bronchial tissue of COPD patients were detected when compared to healthy control subjects [19]. In our previous research, Hsp70 expression was significantly decreased in leukocytes of COPD patients, especially in COPD smokers, but also in healthy smokers in comparison to never-smoking individuals, and we suggested suppressed Hsp70 transcription or its increased release from cells as potential underlying mechanisms that could explain the observed phenomenon [20]. Later, we explored effects of extracellular Hsp70 by employing recombinant human Hsp70 protein (rhHsp70) on human monocytic and bronchial epithelial cellular models (primary cells and cell lines), and we confirmed that rhHsp70 alone, and in combination with cigarette smoke, stimulates TLR2 and/or TLR4 receptors, mitogen-activated protein kinase (MAPK) and/or nuclear factor kappa B (NF-κB) signaling pathways and proinflammatory cytokines release [21,22,23]. Positive associations between eHsp70 and cytokines, as well as other inflammatory markers in circulation, were reported [24,25], and we established the presence of systemic inflammation in our group of COPD patients [26]. Concentration of eHsp70 was assessed in peripheral circulation of COPD patients in only a few studies, but with inconsistent results in comparison to healthy subjects (increased or similar values) [4,16,27]. However, data about eHsp70′s predictive value, and its association with disease severity, are lacking.

We hypothesized that the concentration of eHsp70 in plasma of patients with stable COPD is increased in comparison to healthy individuals, and that it increases with disease severity assessed by the level of airflow obstruction, as well as symptoms and history of exacerbations. In addition, we wanted to investigate the association between eHsp70 concentration and smoking status, as well as its associations with COPD multicomponent indices (BODCAT (BMI, airflow obstruction, dyspnea, CAT score), BODEx (BMI, airflow obstruction, dyspnea, previous exacerbations), CODEx (Charlson’s comorbidity index, airflow obstruction, dyspnea, previous exacerbations) and DOSE (dyspnea, airflow obstruction, smoking status, previous exacerbations)) and lung function parameters. Finally, our aim was to evaluate diagnostic performances of eHsp70.

## 2. Materials and Methods

### 2.1. Participants

There were 137 patients at the stable phase of COPD and 95 healthy individuals matched by age and sex. COPD was diagnosed by a specialist pulmonologist at the Clinical Department for Lung Diseases Jordanovac, University Hospital Centre Zagreb (Zagreb, Croatia), in 2017 and 2018. Patients were in the stable phase of COPD without exacerbations during the last three months, without changes in their therapy regime and without infections in the lower respiratory tract. Health state of control subjects was established based on anamnestic data and normal spirometry test results. Both patients and healthy individuals had to be older than 40 years, could not have any lung disease (except COPD for COPD patients), could not have inflammatory diseases, manifest cardiovascular diseases, acute infections, diabetes with severe complications, severe liver diseases, severe kidney insufficiencies, malignant diseases, transplantations or other ongoing inflammations. All of them signed an informed consent for the scientific research they volunteered for and were introduced to the aims of the research. The research was performed in accordance with the Helsinki Declaration and was approved by the Ethics Committee of University Hospital Centre Zagreb (Approval Protocol Number: 02/21/JG) on 29 August 2014 and by the Ethics Committee for Experimentation of Faculty of Pharmacy and Biochemistry, University of Zagreb (Approval Protocol Number: 251-62-03-14-78) on 10 September 2014. In addition to the diagnosis criterion (forced expiratory volume in one second/forced vital capacity (FEV_1_/FVC) < 0.70) by the Global Initiative for COPD (GOLD), there were classifications of disease severity based on airflow limitation assessed by FEV_1_ measurements (GOLD 1–4 stages) as well as the history of symptoms and exacerbations assessed by the score from the COPD Assessment Test (CAT) (GOLD A–D groups) [28]. All participants reported data about smoking status, so groups of healthy nonsmokers (n = 48), healthy smokers (n = 47), COPD nonsmokers (n = 10), COPD former smokers (n = 90) and COPD smokers (n = 37) were formed. For calculation of multicomponent indices related to COPD assessment, the following data were collected for COPD patients: body mass index (BMI), score obtained from modified Medical Research Council (mMRC) Dyspnea Scale, number of previous exacerbations and Charlson’s comorbidity index. Afterwards, BODCAT (BMI, airflow obstruction, dyspnea, CAT score), BODEx (BMI, airflow obstruction, dyspnea, previous exacerbations), CODEx (Charlson’s comorbidity index, airflow obstruction, dyspnea, previous exacerbations) and DOSE (dyspnea, airflow obstruction, smoking status, previous exacerbations) were calculated [29].

### 2.2. Assessment of Lung Function

Airflow limitation was diagnosed by spirometry on a Master-Screen Pneumospirometer (Jaeger, Wurzburg, Germany), and airflow obstruction was confirmed if FEV_1_/FVC was lower than 0.70 after three acceptable measurements. Furthermore, diffusing capacity for carbon monoxide (DLCO) was measured three times on a Master-Screen PFT Pro (Jaeger, Wurzburg, Germany), as described before [29].

### 2.3. Measurement of eHsp70

Blood samples were collected between 7 and 9 a.m. into tubes with anticoagulant ethylenediaminetetraacetic acid (EDTA) (Greiner Bio-One, GmbH, Kremsmünster, Austria) by venepuncture of a large antecubital vein after overnight fasting [26]. Plasma was separated after centrifugation at 1000× *g* for 15 min at 4 °C and stored immediately at 80 °C until eHsp70 determination. eHsp70 concentration was measured using the AMP’D HSP70 high sensitivity ELISA kit (Enzo Life Science, Farmingdale, NY, USA). All experiments were performed following the manufacturer’s protocol and recommendations, including minimal 1:4 dilution of EDTA plasma samples with assay buffer for matrix interference removal. Concentration of eHsp70 was determined in a randomly chosen sample on all plates and was used for internal validation. Calculation of eHsp70 concentration in samples was performed by a four-parameter logistic curve fitting program within Origin software (OriginLab Corporation, Northampton, MA, USA). The sensitivity or limit of detection of the assay was 0.007 ng/mL, as determined by the manufacturer.

### 2.4. Statistical Analysis

Data were tested for normality by the Kolmogorov-Smirnov test, and all data failed it. Therefore, a nonparametric Mann-Whitney test was performed for analysis between controls and COPD patients. When comparing more than two groups based on different classifications, Kruskal-Wallis one way analysis of variance on ranks with post hoc analysis was used. Categorical variables were tested by Chi-squared test. Spearman Rank Order was performed for testing the correlations between investigated parameters, while assessment of predictive value of eHsp70 was obtained by univariate logistic regression analysis. Statistical tests were run in MedCalc statistical software version 17.9.2. (Ostend, Belgium), and results were considered statistically significant if *p* < 0.05.

## 3. Results

### 3.1. Association of eHsp70 with COPD Severity

**Figure 1:**
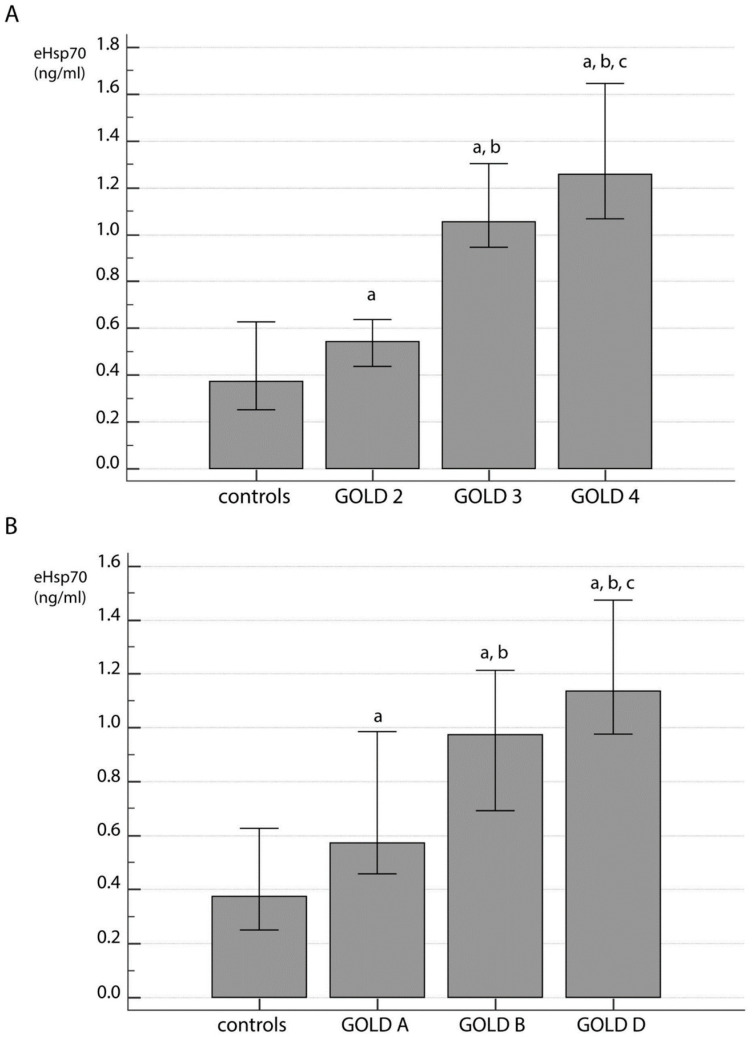
Concentration of eHsp70 in plasma of COPD patients regarding forced expiratory volume in one second (FEV_1_)-based classification by Global Initiative for COPD (GOLD) (**A**) and ABCD classification based on symptoms severity and history of exacerbations (**B**). All data were presented as median with interquartile range. Statistical analysis was performed by Kruskal-Wallis one way analysis of variance on ranks. ^a^ statistically significant increase in eHsp70 concentration in comparison to controls; ^b^ statistically significant increase in eHsp70 concentration in comparison to GOLD 2 (**A**) or GOLD A (**B**); ^c^ statistically significant increase in eHsp70 concentration in comparison to GOLD 3 (**A**) or GOLD B (**B**).

Patients with stable COPD were of similar age as control subjects, and gender distribution was also similar between patients and healthy individuals, while lung function was decreased in the COPD group as expected (Table 1). The concentration of eHsp70 was increased in the plasma of COPD patients (0.98 (0.63–1.29) ng/mL) in comparison to controls (0.37 (0.25–0.63) ng/mL) (*p* < 0.001). Moreover, it was associated with disease severity when COPD patients were subdivided regarding FEV_1_-based airflow limitation (Figure 1A) as well as symptoms severity and history of exacerbations (Figure 1B). eHsp70 showed statistically significant differences regarding GOLD 2–4 stages in comparison to controls (*p* < 0.001) and throughout GOLD A–D groups in comparison to controls (*p* < 0.001). Increasing concentration of eHsp70 successfully distinguished each group of patients regarding both subdivisions.

### 3.2. Influence of Smoking Status on Plasma eHsp70 Concentrations

**Figure 2:**
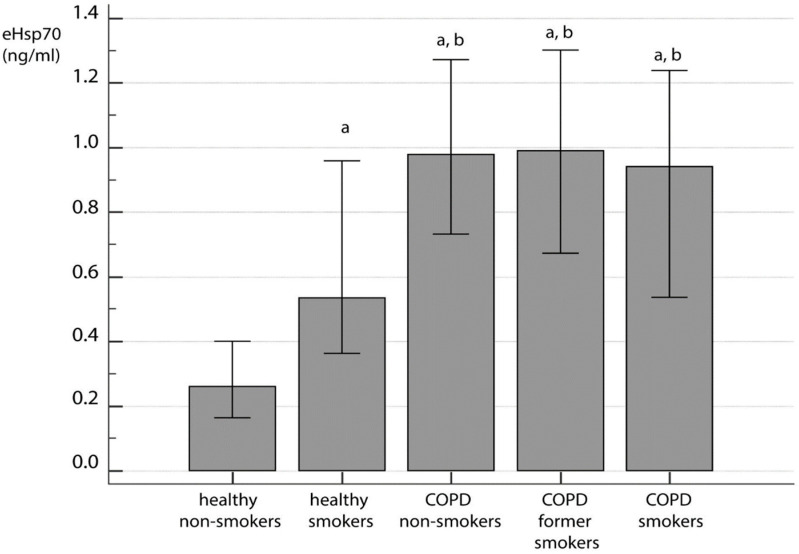
eHsp70 in healthy individuals and COPD patients regarding smoking status. All data were presented as median with interquartile range. Statistical analysis was performed by Kruskal-Wallis one-way analysis of variance on ranks. ^a^ statistically significant increase in eHsp70 concentration in comparison to healthy nonsmokers; ^b^ statistically significant increase in eHsp70 concentration in comparison to healthy smokers.

**Figure 3:**
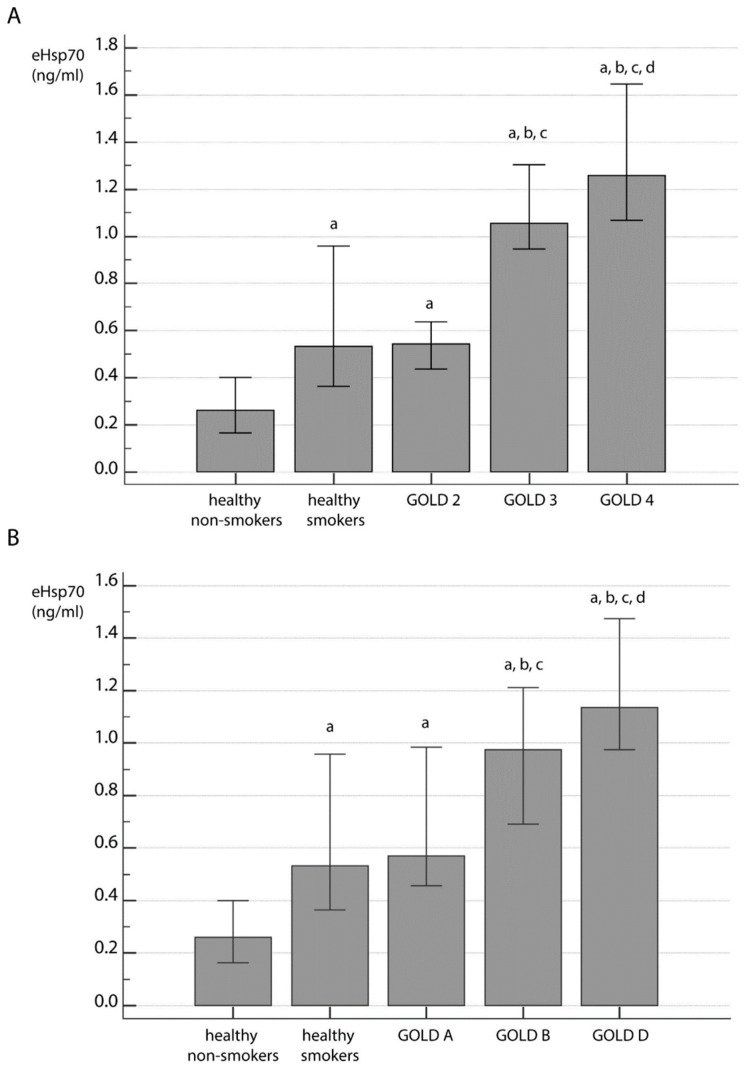
eHsp70 concentration in COPD patients at different stages of FEV_1_-based airflow limitation (**A**) and based on symptoms severity (**B**) compared to healthy subjects regarding their smoking status. All data were presented as median with interquartile range. Statistical analysis between five groups of participants was performed by Kruskal-Wallis one-way analysis of variance on ranks. Significant difference in eHsp70 concentration was observed throughout GOLD 2–4 stages and GOLD A–D groups when healthy individuals were subdivided based on their smoking status (*p* < 0.001). Afterwards, post hoc analysis was performed. ^a^ statistically significant increase in eHsp70 concentration in comparison to healthy nonsmokers; ^b^ statistically significant increase in eHsp70 concentration in comparison to healthy smokers; ^c^ statistically significant increase in eHsp70 concentration in comparison to GOLD 2 (**A**) or GOLD A (**B**); ^d^ statistically significant increase in eHsp70 concentration in comparison to GOLD 3 (**A**) or GOLD B (**B**).

When all participants were compared based on self-reported smoking history, it was observed that there were significant differences in eHsp70 levels between controls and COPD patients (*p* < 0.001). More precisely, COPD patients had increased eHsp70 when compared to both healthy nonsmokers and healthy smokers, yet there was no difference between COPD patients according to their smoking status. However, healthy smokers had higher values of plasma eHsp70 concentrations in comparison to healthy nonsmokers (Figure 2). Additionally, when GOLD 2–4 stages (Figure 3A) and GOLD A–D groups (Figure 3B) were compared to healthy individuals who were grouped according to their smoking status, significant difference in eHsp70 concentration was observed (*p* < 0.001). Healthy smokers had similar levels of eHsp70 in plasma as COPD patients at GOLD 2 stage and those in the GOLD A group, while patients at more advanced disease stages showed increased levels of plasma Hsp70 (Figure 3).

### 3.3. Associations of Lung Function Parameters and COPD Multicomponent Indices With eHsp70

**Figure 4:**
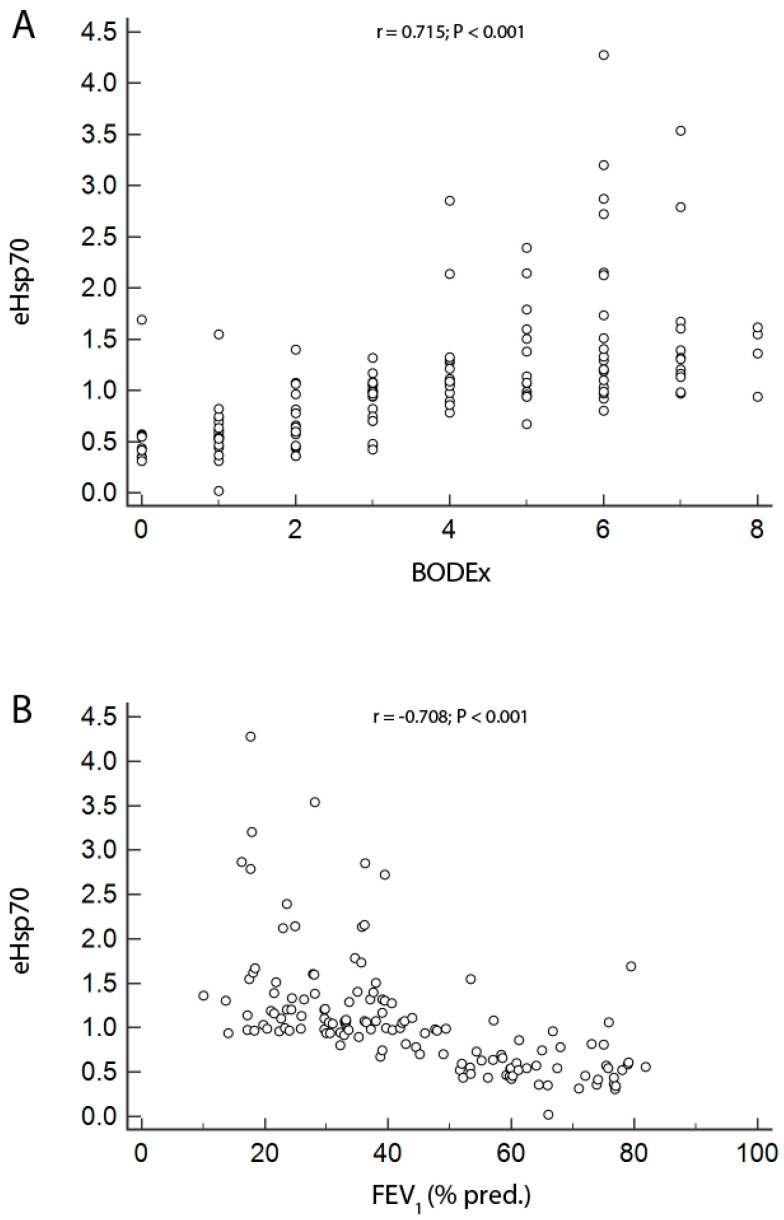
Association of eHsp70 with BODEx (**A**) and FEV_1_ (% pred.) (**B**). r-Spearman’s correlation coefficient; BODEx—BMI, airflow obstruction, dyspnea, previous exacerbations; FEV_1_—forced expiratory volume in one second.

We found no association between eHsp70 levels and age or gender in either COPD patients or controls. However, eHsp70 showed moderate to good positive correlation with COPD multicomponent indices BODCAT, BODEx (Figure 4A), CODEx and DOSE, as well as moderate to good negative correlation with lung function parameters FEV_1_ (L), FEV_1_ (% pred.) (Figure 4B) and FEV_1_/FVC. DLCO and eHsp70 showed to be poorly negatively correlated (*p* < 0.001 for all correlations) (Table 2).

### 3.4. Predictive Performance of eHsp70

Univariate logistic regression analysis with a defined cut-off value of 0.5 showed that eHsp70 had great predictive value with its odds ratio (OR) of 7.63, 95% confidence interval (CI) = 3.68–15.82 and there were 76% cases correctly classified (*p* < 0.001).

## 4. Discussion

COPD is a highly prevalent yet underdiagnosed disease, with increasing morbidity and mortality rates. Complex underlying mechanisms are reflected by diverse clinical presentation and are making this disease challenging for specific diagnosis and therapy. Due to COPD heterogeneity, to personalize the treatment, particular endotype and phenotype should be recognized for each patient.

It is now recognized that inflammation in COPD is not present only at the local level i.e., in lungs and airways, but also at the whole-body level, with persistent systemic inflammation being demonstrated in some patients [30]. A blood sample may be obtained in a relatively noninvasive way which makes it easily accessible. Therefore, searching for a good peripheral blood diagnostic, prognostic, predictive biomarker and/or biomarker of disease severity in any disease is recommendable, especially in complex and heterogeneous diseases.

The potential pathogenetic role of eHsp70 is still quite obscure. However, it seems to be associated with an eHsp70 immunomodulatory function. Immune cells can recognize eHsp70, which initiates signal transduction and results in the release of cytokines. Moreover, crosstalk with TLRs activates proinflammatory signals which result in promoting and prolonging chronic inflammation [31]. Also, inflammation-related outcomes of eHsp70 might be executed by NLRP3 inflammasome activation [32]. Besides COPD, eHsp70 seems to be implicated in other respiratory diseases, such as asthma, with a similar pathogenetic background. It was reported that plasma eHsp70 was increased in asthmatic patients compared to healthy individuals [3], as well as in pregnant asthmatics, in comparison to healthy pregnant women [33]. Some studies also detected elevated concentrations of Hsp70 in the sera of lung cancer patients [34,35].

In this study, we assessed the concentration of Hsp70 in the peripheral blood of patients with stable COPD, and association with disease characteristics defined by spirometry and clinical presentation. eHsp70 was significantly elevated overall in COPD patients compared to healthy subjects. This increase was related to the degree of airflow limitation as well as symptoms burden and history of exacerbation. eHsp70 concentrations were the highest in GOLD 4 stage and GOLD D group. It is also important to emphasize that eHsp70 was elevated even in GOLD A and GOLD 2 (which, in clinical practice, is often the lowest GOLD stage) compared to the overall control group. To the best of our knowledge, this is the first study that has assessed eHsp70 concentrations in COPD patients subdivided by the GOLD ABCD classification, and the first to show differences in eHsp70 levels according to GOLD stages.

Dong et al. reported that the expression of intracellular Hsp70 was closely related with COPD severity, and was higher in GOLD 2, 3 and 4 stages compared to the GOLD 1 stage [17]. However, the association of extracellular Hsp70 with disease severity has not yet been shown for COPD, but demonstrated for some other diseases, namely asthma, chronic heart failure and rheumatoid arthritis [3,36,37].

In the present study, when participants of the control group were subdivided according to their smoking status, patients belonging to the GOLD 2 and GOLD A subgroups had higher eHsp70 levels than never-smoking individuals, but similar eHsp70 levels as smokers with normal lung function. Therefore, it could be suggested that so-called healthy smokers might be more susceptible to altered inflammatory responses provoked by eHsp70 being a danger signal to the immune system, and some of them might even develop COPD in the future. This assumption should be tested in future studies. In addition, as only 20% of smokers develop COPD, a specific individual genetic makeup seems to be a determined for the disease.

By searching the literature, we found only three studies that assessed Hsp70 concentration in the blood of COPD patients [4,16,27]. However, in addition to being performed on significantly lower number of participants compared to our study, there are some concerns regarding their sample and ELISA kit selection. Reported concentrations of eHsp70 are very dependent on the matrix in which it is measured. Whitham and Fortes demonstrated that eHsp70 concentrations were the highest in EDTA plasma. However, values in heparinized plasma were somewhat lower, while the lowest eHsp70 levels were measured in serum, and they hypothesized that this was due to the binding of eHsp70 to the aggregated clotting proteins in serum. Therefore, they recommended EDTA plasma as a sample of choice in future investigations [8]. This is important for studies with healthy participants at rest as their eHsp70 values tend to be low, as well as for the studies with elderly subjects since their eHsp70 concentrations are significantly lower than in young individuals [25].

As already mentioned, eHsp70 in COPD patients was assessed in serum [4,27] or heparinized plasma [16]. However, although Ünver et al. used serum samples, the measurement of eHsp70 was performed by an adopted ELISA kit that was not entirely appropriate for blood as a matrix, and this could be the reason for the extremely high eHsp70 values obtained in the study [4]. Hacker et al. also used an adopted ELISA kit that was specific for intracellular Hsp70 determination in cell lysates [27]. Limitations of these assays were caused by the lack of optimization for biological fluids such as blood. On the other hand, Cui et al. selected a proper ELISA kit which was validated for serum and EDTA plasma, but they chose heparinized plasma as the sample for eHsp70 measurement, which might be the reason for obtaining higher eHsp70 values [16]. In the present study, we used EDTA plasma and an ELISA kit that was more sensitive and could detect lower eHsp70 concentrations compared to other ELISA kits also validated for EDTA plasma and serum [38]. With this choice of sample and kit we were able to detect eHsp70 in each study participant (controls and patients).

In this study, we obtained positive associations between eHsp70 and multicomponent COPD indices (BODCAT, BODEx, CODEx, DOSE) that reflect airflow obstruction, smoking status, symptoms and history of exacerbations, which are all important in the assessment of the patients’ overall condition. We also obtained significant negative associations between eHsp70 and lung function parameters, and this was only reported for the expression of intracellular Hsp70 in COPD patients with good correlation for FEV_1_/FVC and poor correlation for FEV_1_ (% pred.) [17]. Finally, eHsp70 was shown to have a good predictive characteristic with its OR of 7.63 (95% CI = 3.68–15.82).

Although we presented some novel and interesting results, our study had some limitations. It did not include COPD patients from the GOLD C group or the GOLD 1 stage. However, in clinical practice COPD patients belonging to the GOLD 1 group rarely contact their physician due to very mild symptoms, and the GOLD C category of patients is also very rare as they do not have many symptoms and are not usually frequent exacerbators. A larger number of participants should be recruited in further studies, and a longitudinal study design should be considered.

## 5. Conclusions

This study demonstrated that eHsp70 concentrations were increased in EDTA plasma of COPD patients in the stable phase of the disease when compared to healthy subjects, and its levels were associated with airflow limitation as well as symptoms burden and history of exacerbations. Smokers with normal lung function had significantly higher eHsp70 values than healthy never-smokers, and chronically elevated eHsp70 might contribute to the development of some pathologies in the future, including COPD, in some genetically or otherwise susceptible healthy smokers. We suggest that eHsp70 has a potential to become a new biomarker in COPD assessment, and its evaluation in healthy smokers might also merit further investigation.

## Figures and Tables

**Table 1:** Basic characteristics and spirometry parameters of all participants. Age was shown as median with minimum and maximum, and gender as absolute number. All other data were presented as median with interquartile range. Data were tested by Chi-squared or Mann-Whitney test.

Parameter	Controls n = 95	COPD Patients n = 137	*p*-Value
age	64 (46–83)	65 (44–86)	0.073
gender male/female	49/46	86/51	0.118
FEV_1_ (L)	2.60 (2.12–3.19)	1.08 (0.78–1.57)	<0.001
FEV_1_ (% pred.)	93 (86–104)	39 (28–60)	<0.001
FVC (L)	3.35 (2.77–4.16)	2.28 (1.81–2.77)	<0.001
FEV_1_/FVC (%)	81 (77–88)	48 (41–58)	<0.001

COPD–chronic obstructive pulmonary disease; FEV_1_—forced expiratory volume in one second; FVC—forced vital capacity.

**Table 2:** Spearman Rank Order analysis was performed between eHsp70 and COPD multicomponent indices as well as lung function parameters.

Parameter	Spearman’s Correlation Coefficient, r	*p*-Value
BODCAT	0.712	<0.001
BODEx	0.715	<0.001
CODEx	0.710	<0.001
DOSE	0.672	<0.001
FEV_1_ (L)	−0.658	<0.001
FEV_1_ (% pred.)	−0.708	<0.001
FEV_1_/FVC	−0.644	<0.001
DLCO	−0.479	<0.001

BODCAT—BMI, airflow obstruction, dyspnea, score from COPD assessment test (CAT); BODEx—BMI, airflow obstruction, dyspnea, previous exacerbations; CODEx—comorbidities (Charlson’s index), airflow obstruction, dyspnea, previous exacerbations; DOSE—dyspnea, airflow obstruction, smoking status, previous exacerbations; FEV_1_—forced expiratory volume in one second; FVC—forced vital capacity; DLCO—diffusing capacity for carbon monoxide. Previous exacerbations are defined as the number of exacerbations in the previous year.
